# Expanding the Reach of Membrane Protein–Ligand Interaction Studies Through the Integration of Mass Spectrometry and Membrane Mimetics

**DOI:** 10.1002/pmic.70057

**Published:** 2025-10-15

**Authors:** Jonathon C. Lambos, Ashim Bhattacharya, Mohammed Al‐Seragi, Franck Duong van Hoa

**Affiliations:** ^1^ Department of Biochemistry and Molecular Biology Faculty of Medicine Life Sciences Institute University of British Columbia Vancouver British Columbia Canada

**Keywords:** detergents, mass spectrometry, membrane mimetics, membrane proteins, protein–ligand interaction

## Abstract

Mass spectrometry (MS) offers robust, label‐free approaches for characterizing ligand–protein interactions through two main strategies: affinity‐based and stability‐based assays. However, their application to membrane proteins (MPs)—a major class of drug targets—has been limited by challenges such as structural complexity, low native expression, incomplete trypsin digestion, and poor compatibility with detergent‐based MS protocols. Recent progress has advanced the field along two complementary fronts. First, innovations in MS methodology, including native MS, nativeomics, solution‐phase thermochemistry, and ion mobility‐mass spectrometry (IM‐MS), have improved the ability to preserve intact assemblies, capture co‐bound lipids and ligands, and resolve conformational and energetic landscapes of MPs. Second, advances in MP solubilization and stabilization, through tailored detergent architectures, MS‐compatible detergents, and membrane mimetic (MM) systems—such as nanodiscs, peptidiscs, and styrene–maleic acid (SMA) polymers—have created more native‐like environments that maintain functional conformations and ligand‐binding sites, enabling integration of MPs into high‐throughput MS platforms for ligand screening. This review outlines key affinity‐ and stability‐based MS approaches for MPs and highlights how advances in MS methodology and solubilization strategies are extending their scope, positioning MS and MM as an increasingly powerful platform for high‐throughput discovery of MP–ligand interactions.

**Abbreviations**
β2‐AR: β2‐adrenergic receptor
A_2_AR: A_2_A adenosine receptor
AS‐MS: affinity selection mass spectrometry
ATP: adenosine triphosphate
AβO: amyloid‐β oligomer
CaSR: calcium‐sensing receptor
CCS: collision cross section
CETSA: cellular thermal shift assay
CSC: cell surface capture
CS‐TPP: cell‐surface thermal proteome profiling
DARTS: drug affinity responsive target stability
DDM: dodecyl beta maltoside
DeFrND: detergent‐free nanodisc
DIA: data‐independent acquisition
DSF: differential scanning fluorimetry
ELISA: enzyme‐linked immunosorbent assay
GLP‐1R: GLP‐1 receptor
GPCRG: protein coupled receptor
IC_50_
: half maximal inhibitory concentration
IM‐MS: ion mobility‐mass spectrometry
iTSA: isothermal shift assay
*K*_d_
: dissociation constant
*K*_off_
: dissociation rate constant
LC‐MS/MS: liquid chromatography–tandem mass spectrometry
LiP‐MS: limited proteolysis‐mass spectrometry
LMNG: lauryl maltose neopentyl glycol
MM: membrane mimetic
MM‐TPP: membrane mimetic thermal proteome profiling
MP: membrane protein
MS: mass spectrometry
MS/MS: tandem mass spectrometry
MSBA: mass spectrometry binding assay
MSP: membrane scaffold protein
nAChR: nicotinic acetylcholine receptor
OGD: oligoglycerol detergent
PELSA: proteome‐wide elution profile learning for small‐molecule activity
SEC: size exclusion chromatography
SMA: styrene–maleic acid
SMALP: styrene maleic acid lipid particle
SPA: scintillation proximity assay
SPROX: stability of proteins from rates of oxidation
STPP‐UP: single‐tube thermal proteome profiling with uniform progression
TALiP‐MS: thermostability‐assisted limited proteolysis mass spectrometry
*T*_m_
: melting temperature
TMT: tandem mass tag
TMT‐DDA: tandem mass tag‐based data‐dependent acquisition
TPP: thermal proteome profiling

## 1 Introduction

Mass spectrometry (MS), long regarded as the gold standard for protein identification, has evolved into a powerful tool for mapping protein–ligand interactions. With the advent of label‐free detection formats, MS now occupies a critical intersection between structural proteomics and drug discovery. This evolution did not occur abruptly but unfolded gradually, paralleling a broader shift in proteomics from merely cataloguing proteins to probing their functional roles within the cellular environment [1].

In protein–ligand studies, this transition toward functional interrogation has been driven by two main classes of MS‐based methodologies: affinity‐based and stability‐based approaches, named according to their underlying mechanisms [2]. Affinity‐based techniques, such as affinity selection mass spectrometry (AS‐MS) and mass spectrometry binding assays (MSBAs), directly capture protein–ligand complexes, which are then analyzed via liquid chromatography–tandem mass spectrometry (LC‐MS/MS) to reveal bound ligands [3, 4]. This workflow, despite being technically challenging, preserves native interactions and provides straightforward readouts. Modernization in high‐throughput formats has dramatically scaled these assays, with pooled AS‐MS screens now encompassing libraries of over 20,000 compounds [5]. Stability‐based approaches, by contrast, infer binding events indirectly through changes in protein conformation. Methods such as stability of proteins from rates of oxidation (SPROX), thermal proteome profiling (TPP), and limited proteolysis‐mass spectrometry (LiP‐MS) detect ligand engagement by measuring thermal shifts, oxidation rates, or altered proteolytic cleavage patterns [6, 7, 8]. These methods ask not “what binds,” but “is binding altering the protein?” In other words, thermal shifts or altered cleavage patterns serve as proxies for ligand engagement [9]. TPP, in particular, has proven effective for profiling both on‐ and off‐target interactions in whole‐cell lysates, offering systems‐level insights into compound activity [10, 11, 12, 13, 14].

Despite these advances, MPs, which represent nearly 70% of the druggable proteome, remain underrepresented in high‐throughput MS screening campaigns [15, 16]. This exclusion is due to a set of enduring biochemical challenges: MPs are often present in low abundance, structurally complex, embedded in lipid bilayers that resist solubilization, and are prone to incomplete digestion due to the burial of their transmembrane segments. In consequence, amphipathic molecules like detergents have been employed to extract MPs from the lipid bilayer, and several MP discoveries have been achieved this way, including landmark structural elucidation of the rhodopsin G‐protein coupled receptor (GPCR) receptor and the multi‐drug exporter ABCB1, among others [17, 18]. However, detergents have been less successful in preserving ligand–MP interactions for MS‐based interrogation. Detergents easily destabilize native protein conformations, disrupt ligand‐binding sites, and interfere with downstream MS analysis [16, 19]. Balancing solubilization with preservation of native structure and functional lipid–protein interactions, which often act as essential cofactors, remains a major challenge [20]. This issue is especially pronounced for mammalian MPs, which are highly dynamic and whose structural integrity and functional activity are tightly coupled to the surrounding lipid environment [21]. Nonetheless, emerging studies suggest that with suitable adaptations, MS‐based methods can be extended to MPs, spurring continued innovation in this area [10, 22, 23, 24].

**TABLE 1:** Summative comparison of key affinity‐ and stability‐based MS approaches and application to MPs.

	Method	Principle	Chromatography	Throughput	Strengths	Limitations	Feasibility with membrane proteins	Publications with membrane proteins
Affinity‐based	Affinity‐selection mass spectrometry (AS‐MS)	Separation of bound versus unbound ligands using an immobilized protein of interest on a matrix (e.g., beads), followed by MS detection of retained ligands. Separation typically occurs via ultrafiltration, ultracentrifugation, or size exclusion chromatography (SEC).	Requires a tagged protein and a matrix for the protein to be immobilized on. Separates ligands via size exclusion chromatography	Screens small or large compound libraries against a single protein target.	Label‐free and direct detection of ligand binding. Suitable for early‐phase ligand screening. Only strong ligand–protein binders are retained, minimizing false positives.	Requires a large amount of purified protein. May suffer from non‐specific or background binding. No structural or functional insight. Incompatible with ligands that ionize poorly in MS.	Compatible with detergent‐solubilized or mimetic‐reconstituted membrane proteins. Since ligands are analyzed (not the protein), detergent cleanup is not required. Less suitable for low‐abundance membrane proteins due to high protein input requirements.	[5, 22, 25, 26]
	Mass spectrometry binding	Detection of ligand–target interactions via changes in the mass or retention of an unlabeled reporter ligand in the presence of a compound library, analyzed by MS.	Not required	Screens a compound library against a known protein target using competitive binding to a reporter ligand.	Enables quantification of ligand kinetics and specificity. Distinguishes on‐target from off‐target interactions. No chromatography required. Label‐free readout.	Requires a well‐characterized reporter ligand with a known binding site. Limited to targets with established ligands. No functional or structural readout. Incompatible with ligands that exhibit poor or inconsistent MS ionization.	Compatible with detergent‐solubilized or mimetic‐reconstituted membrane proteins. Since ligands are analyzed rather than the protein, detergent cleanup is not required. Less suitable for low‐abundance membrane proteins due to high protein input requirements.	[27, 28, 29]
Stability‐based	Stability of proteins from rates of oxidation (SPROX)	Measures protein stability by monitoring methionine oxidation following protein incubation with a chemical denaturant and hydrogen peroxide.	Requires proteolytic digestion and peptide cleanup (e.g., C18 stage tip) before LC‐MS/MS analysis.	Screens ligand binding to individual proteins or across complex proteomes.	Compatible with complex lysates. Detects ligand‐induced stability changes. Supports isobaric labeling for multiplexing.	Only applicable to methionine‐containing peptides. Prone to oxidative side reactions. Does not allow real‐time measurement.	Challenging for membrane proteins due to limited solvent‐exposed methionine. Requires detergent cleanup or membrane mimetic systems, which may restrict methionine accessibility and obscure oxidation‐based readouts.	[23]
	Thermal proteome profiling (TPP)	Measures ligand‐induced shifts in protein thermal stability across the proteome using MS‐based quantification after heat‐induced denaturation.	Requires proteolytic digestion and peptide cleanup (e.g., C18 stage tips) before MS analysis.	Screens ligand binding across global proteome or targeted subproteomes.	Global and unbiased profiling. compatible with complex lysates. supports isobaric labeling for multiplexing.	Requires thermostable proteins. Susceptible to off‐target or metabolite‐induced shifts. High sample and MS time demand unless multiplexed with TMT. May suffer from reproducibility issues across replicates.	Challenging for detecting ligand interactions with membrane proteins, especially in non‐solvent‐exposed regions obscured by detergents or mimetics. Membrane proteins often have low endogenous abundance and may require detergent cleanup or mimetic‐based reconstitution for MS compatibility.	[10, 11, 13, 14, 30, 31]
	Limited proteolysis mass spectrometry (LiP‐MS)	Detects ligand binding by monitoring shifts in protease‐accessible regions of proteins caused by ligand‐induced conformational changes.	Requires proteolytic digestion and peptide cleanup (e.g., C18 stage tips) before MS analysis.	Screens ligand binding across individual proteins or proteomes.	Provides site‐level conformational insight. Applicable to complex mixtures. supports isobaric labeling for multiplexing.	Highly sensitive to protease selection and digestion conditions. Proteome coverage can be limited without extensive MS acquisition.	Challenging for membrane proteins due to poor protease accessibility and resistance of transmembrane domains to digestion. Requires detergent cleanup or membrane mimetic systems to expose accessible regions for analysis.	

As part of these innovations, MS methodology remains a key area of development for expanding ligand–MP studies. Native MS preserves intact assemblies, allowing detection of ligand‐bound MP‐complexes [32]. Nativeomics combines native MS with proteomic, lipidomic, and metabolomic analyses to identify MPs and characterize their bound partners [33]. In addition, MP assemblies can also be released from native or reconstituted membranes, enabling detection of complexes with bound ligands [34]. Solution‐phase thermochemistry characterizes ligand binding by measuring changes in collision‐induced protein unfolding, while ion mobility‐mass spectrometry (IM‐MS) resolves conformational dynamics to reveal how ligands stabilize or destabilize MP assemblies [35]. Finally, advances in detergent chemistry have improved the balance between solubilization and structural preservation, enabling MS‐compatible workflows for challenging MPs [36, 37].

Parallel to these technological developments, researchers are exploring the use of alternative membrane mimetics (MMs) to stabilize MPs in aqueous environments while preserving their native conformations. These MMs, either protein‐, peptide‐, or polymer‐based, help support protein stability but also make MPs more amenable to diverse analytical workflows compared to detergents. Among these, nanodiscs, styrene–maleic acid (SMA) copolymers, and the peptidisc have been adopted with success. Nanodiscs create a bilayer‐like environment by reconstituting MPs into disk‐shaped lipid patches stabilized by two membrane scaffold proteins (MSPs) derived from apolipoprotein A [38]. SMA copolymers, on the other hand, extract MPs directly from native membranes by encircling patches of the lipid bilayer, forming SMA lipid particles (SMALPs) [39]. Finally, the peptidisc, a multicopy and lipid‐free peptide scaffold, characterized by its “one‐size‐fits‐all” design, offers broad compatibility with a variety of MPs [24, 40].

As MS‐based ligand discovery workflows continue to gain sensitivity and throughput, advances in MS methodology, detergent chemistry, along the integration of MMs provide not only technical solutions but also a broader conceptual framework for re‐engaging a class of proteins long sidelined by solubility barriers. Together, these technologies offer a promising route for incorporating MPs into drug discovery pipelines. This review, therefore, aims to address three fundamental questions: (i) How are affinity‐ and stability‐based MS approaches applied to MP–ligand interactions, and what are the major challenges? (ii) What advances in MS methodology and detergent chemistry have improved the preservation of native MP interactions? And (iii) how can integrating MMs with MS overcome traditional obstacles in MP–ligand studies?

## 2 Affinity Based Methods

### 2.1 Affinity Selection Mass Spectrometry

**FIGURE 1:**
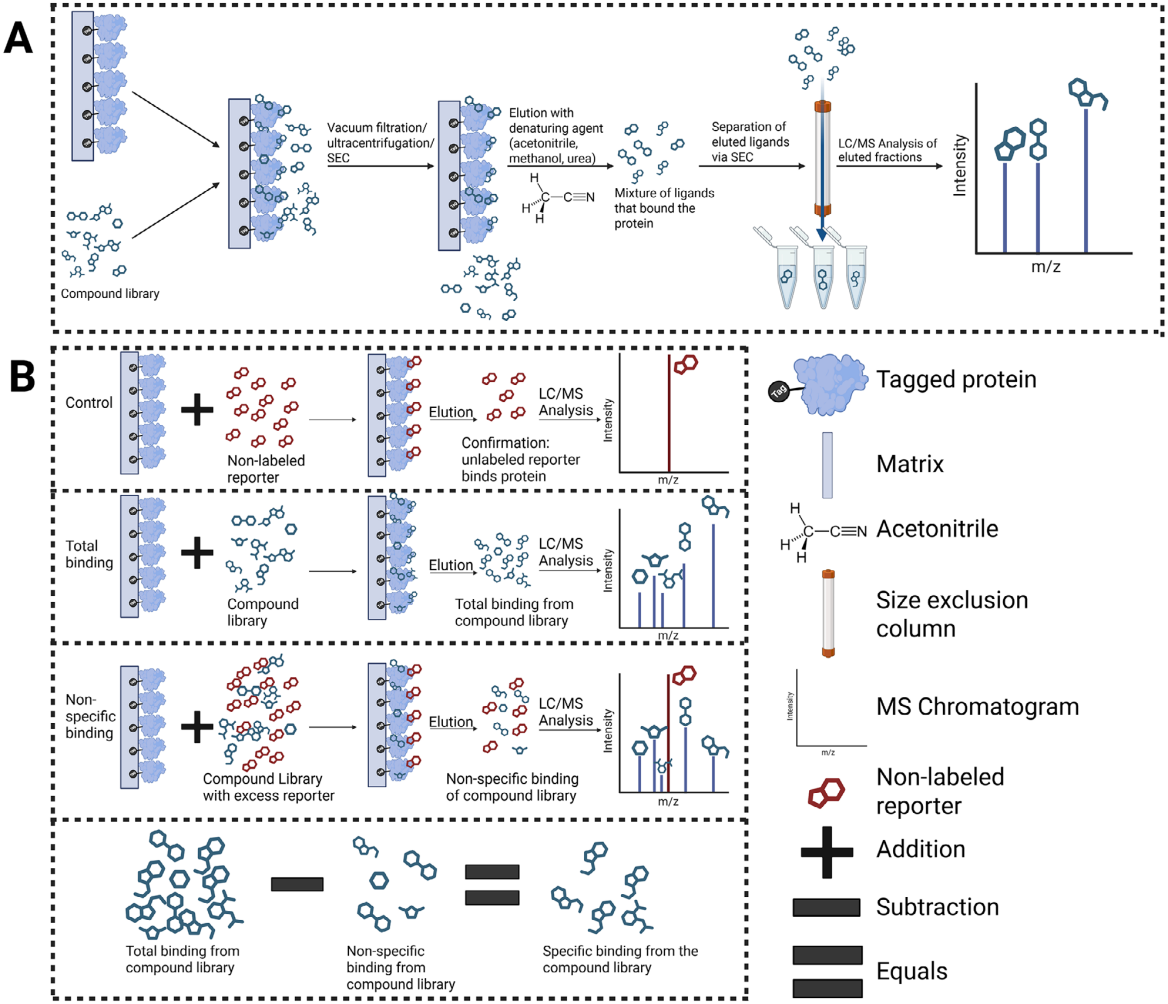
Schematic overview of affinity‐based mass spectrometry approaches for ligand–protein interaction discovery. (A) Schematic of affinity‐selection mass spectrometry (AS‐MS). A small molecule library is incubated with a tagged target protein immobilized on a filter or a matrix (e.g., Ni‐NTA for His‐tagged proteins). Unbound and weakly bound compounds are removed via wash steps, vacuum filtration, ultracentrifugation, or size exclusion chromatography (SEC). Bound ligands are then eluted using a denaturing solvent such as acetonitrile, methanol, or urea. The ligand mixture is further separated by SEC, and individual fractions are analyzed by LC/MS to identify protein‐binding compounds. (B) Schematic of mass spectrometry binding assays (MSBA). This approach uses three parallel conditions to distinguish specific from non‐specific binders. First, a known unlabeled reporter ligand is incubated with the protein‐matrix to confirm target engagement via LC/MS (control). Second, the test compound library is incubated with the protein to capture all potential binders (total binding). Third, the compound library is tested with the protein in the presence of excess reporter ligand, which competitively blocks target sites so that only non‐specific interactions from the library compounds are detected (non‐specific binding). Subtraction of the non‐specific binding from the total binding reveals the specific binders within the compound library. Key reagents and steps are indicated in the schematic, including ligand addition, elution, and MS readouts.

AS‐MS exemplifies the core strength of affinity‐based screening, that is, capturing protein–ligand complexes in solution and identifying bound molecules directly by mass [3]. This approach has become increasingly valuable in early‐stage drug discovery, where the combination of high analytical resolution and throughput is critical for effective screening [5, 22, 25, 26, 41]. In a typical AS‐MS workflow, a purified protein of interest is immobilized, often via an epitope tag, on a solid support such as magnetic or agarose beads (Figure 1A). After incubation with a ligand mixture, unbound and weakly bound ligands are removed using methods like vacuum filtration, ultrafiltration, or size exclusion chromatography (SEC). The remaining ligands are eluted with denaturants (e.g., organic solvents or detergents) and identified via LC‐MS/MS, with mass‐to‐charge ratios and retention times serving as key ligand identifiers [5, 25, 26, 41]. This method enables direct screening of complex mixtures, such as compound libraries or natural product extracts, without requiring ligands to be labeled or individually purified beforehand [3, 41].

AS‐MS performs with high sensitivity and precision when applied to soluble proteins. However, extension of the workflow to MPs presents significant biochemical and technical challenges, not due to the method itself, but due to the instability and heterogeneity of MPs when removed from their native lipid environments. For many multipass MPs (e.g., GPCRs), detergent purification often induces structural distortion or conformational bias, which directly impacts ligand detection. Nonetheless, adaptations of AS‐MS to detergent‐solubilized MPs have shown promise. For example, Zhang et al. used AS‐MS on dodecyl beta maltoside (DDM)‐purified 5‐HT2C receptors immobilized on magnetic beads, identifying aporphine alkaloid compound 1857, a G protein‐biased agonist that selectively activates 5‐HT2C over 5‐HT2A and 5‐HT2B, with implications for weight loss [26]. Despite the potential of these approaches, studies have noted a tendency for ligand discovery to favor antagonists over functionally relevant modulators. This bias can arise when purified MPs are stabilized in a particular conformation, preferentially accommodating ligands with specificity for that specific conformation, thereby skewing the screen toward one class of ligands. The risk of conformational bias was illustrated by Lu et al., who screened a thermostabilized A_2_A adenosine receptor (A_2_AR) against 1100 compounds. They identified Fg754, a negative allosteric modulator. However, their use of a thermostabilized A_2_AR variant likely restricted the discovery of ligands that bind the receptor in native conformations [5]. Thermostabilization, while beneficial for expression and handling, often limits receptor dynamics that are critical for engaging certain classes of ligands.

Recognizing the limitation of conformation bias, researchers have shifted toward screening in native membrane environments. Qin et al. developed an AS‐MS workflow using crude insect cell membranes containing GLP‐1 receptors (GLP‐1Rs) as bait. This setup preserved native conformation and enabled in situ ligand binding, followed by filtration and LC‐MS identification. The study isolated 18 ligands, including four novel positive allosteric modulators. Notably, these ligands failed to bind a thermostabilized GLP‐1R mutant, underscoring the importance of preserving native MP dynamics during screening [25, 42].

Although such advances are encouraging, AS‐MS faces inherent limitations that constrain its broader application to MPs. First, detection sensitivity depends heavily on the MS platform, and ligands with poor or inconsistent ionization, even if tightly bound, may go undetected [27]. Additionally, protein–ligand complexes can dissociate during washing or separation steps. Protocol optimizations (e.g., adjusting protein concentration, ligand ratios, buffer conditions) can reduce these losses, but do not fully resolve the challenges unique to MPs in detergent systems [5, 25, 43]. The challenge extends to input requirements as well. AS‐MS often demands substantial quantities of purified protein, which poses a logistical bottleneck for MPs since overexpression systems are generally less effective for MPs than for soluble proteins [27, 43]. Collectively, these factors contribute to the underrepresentation of MP targets in AS‐MS campaigns.

### 2.2 Mass Spectrometry Binding Assays

Unlike AS‐MS, which directly detects ligand binding, MSBAs are a competitive technique that utilizes a non‐labeled, MS‐detectable reporter ligand, referred to as a marker. Rather than measuring the presence of bound ligands, the primary readout in MSBA is the extent to which test compounds displace the pre‐bound marker from its binding site [27, 43, 44]. In a typical MSBA workflow, a compound library is divided into sub‐libraries and screened in parallel under two conditions: one to measure total marker binding, and the other including an excess of unlabeled competitors to determine specific binding (Figure 1B). Following incubation, unbound compounds are removed through a washing step, and the remaining bound ligands are eluted and identified by LC‐MS/MS [43, 45]. This washing step is a critical assay parameter and largely depends on the affinity of the reporter ligand. High‐affinity markers allow for stringent washing via filtration, while weaker interactions may require gentler separation methods such as density‐gradient centrifugation.

Early studies demonstrated that MSBA could be conducted in membrane preparations without detergent solubilization. For example, Sichler et al. employed filtration using a deuterium‐labeled reporter ligand, [^2^H6]MB327, to investigate nicotinic acetylcholine receptor (nAChR) interactions. In this study, nAChRs were presented in their native conformation using membrane vesicles prepared from *Torpedo californica* electroplaque tissue [28]. In a related effort, Chen et al. (2017) expressed the human BLT1 receptor in HEK293 cells and developed the MSBA workflow using membrane preparations. They characterized two reporter ligands with high affinity and strong MS signals, ensuring precise competition‐based measurements. Their assays were sufficiently sensitive to provide kinetic parameters, including ligand–receptor dissociation rate (*K*_off_) and constants (*K*_d_) [29]. Subsequently, Gabriel et al. built on this membrane‐based approach by integrating MSBA with AS‐MS in a two‐step workflow. Using the GAT1 neurotransmitter transporter expressed in HEK293 membranes, they first identified candidate binders from a compound library using MSBA. These candidates were then validated via AS‐MS [27]. This dual strategy allowed researchers to leverage MSBA for affinity and kinetic insights, and AS‐MS for structural validation of ligand–protein binding.

However, these MS assays are not without limitations. They fundamentally depend on the availability of a well‐behaved marker ligand, one with high specificity, favorable ionization, and stable binding. Without such a reference, competitive displacement cannot be measured [43, 46]. Moreover, they only detect novel ligands that bind to the same site as the marker ligand. Additional complications arise in membrane‐rich preparations, where non‐specific interactions between lipophilic compounds and bilayer components can elevate background signal and reduce assay fidelity, as off‐target partitioning may obscure true displacement [42].

## 3 Stability‐Based Methods

### 3.1 Stability of Proteins From Rates of Oxidation

**FIGURE 2:**
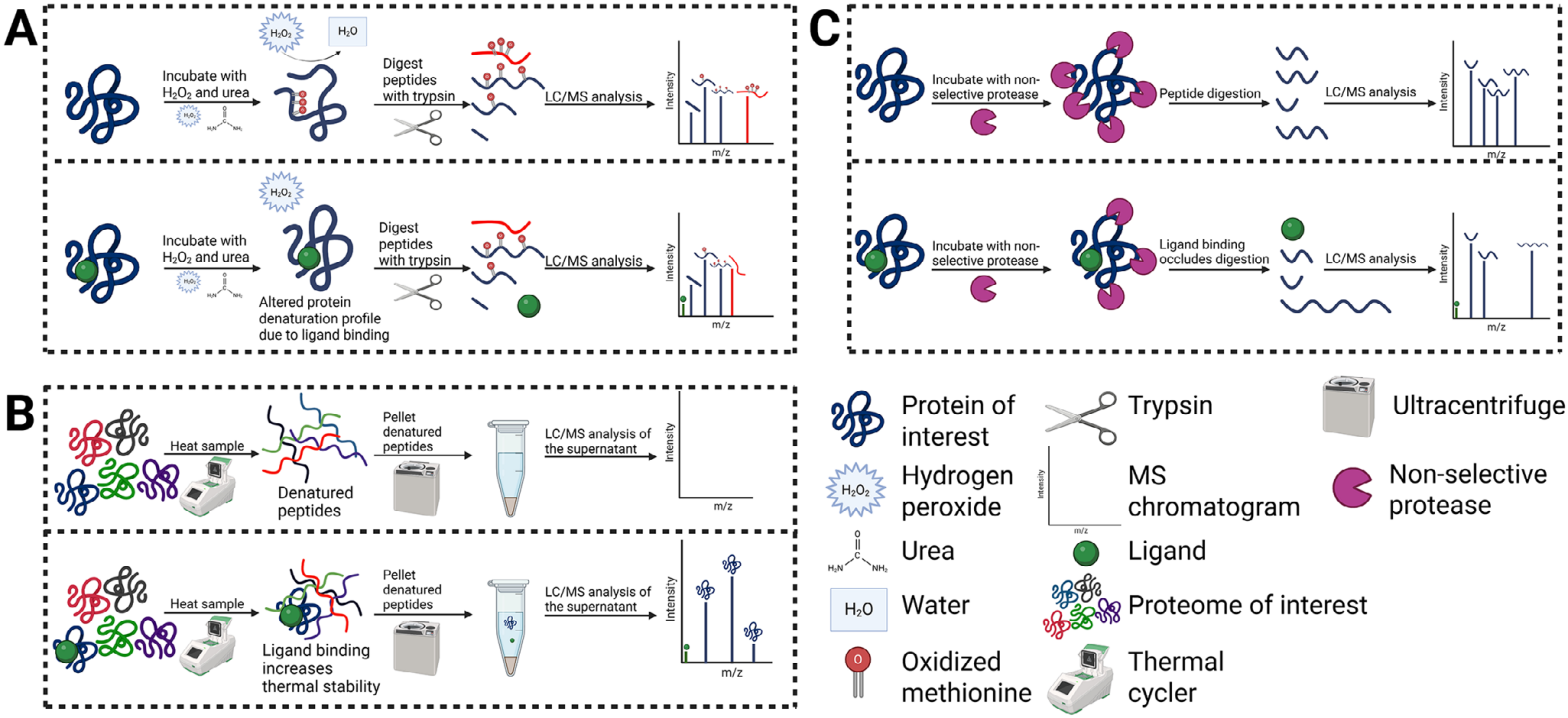
Schematic overview of stability‐based mass spectrometry approaches for the discovery of ligand–protein interactions. (A) Schematic of the stability of proteins from rates of oxidation (SPROX). The protein of interest is incubated with hydrogen peroxide and a denaturant such as urea, in both control and ligand‐treated conditions. In the absence of ligand, protein unfolding exposes buried methionine residues, which are subsequently oxidized. After digestion with trypsin, peptides are analyzed by LC/MS, with methionine oxidation‐induced mass shifts reflecting the protein's denaturation profile. In the presence of ligand, binding can alter the stability of the protein of interest, altering its unfolding trajectory and resulting in a distinct methionine oxidation pattern. Comparing these profiles allows for the identification of ligand‐induced stabilization events. (B) Schematic of thermal proteome profiling (TPP). The proteome of interest is heated at different temperatures using a thermal cycler, resulting in the denaturation and thus aggregation of thermally unstable proteins. The sample is then centrifuged, pelleting the denatured proteins while the non‐denatured proteins remain in the supernatant. Subsequently, the supernatant is analyzed by LC/MS, detecting the non‐denatured proteins. In the ligand‐treated condition, ligand binding can alter the thermal stability of specific proteins, leading either to stabilization that prevents denaturation or to destabilization that promotes unfolding, aggregation, and subsequent pelleting during centrifugation. These changes are detected by LC/MS, and differences in protein abundance between conditions enable the inference of ligand–protein interactions. (C) Schematic of limited proteolysis mass spectrometry (LiP‐MS). The protein of interest is treated with a non‐selective protease in both control and ligand conditions. In the absence of ligand, accessible regions are digested, generating characteristic peptide digestion fragmentation patterns detected by LC/MS. Ligand binding can occlude protease cleavage sites, leading to altered digestion patterns. Comparative analysis of these peptide profiles reveals ligand‐induced conformational protection and identifies binding regions.

SPROX is a chemical strategy for detecting ligand‐induced changes in protein stability by monitoring methionine oxidation as a structural probe. Under denaturing conditions (e.g., urea or guanidine hydrochloride), buried methionines become exposed to hydrogen peroxide and oxidize to methionine sulfoxide (+16 Da), which is readily detected by MS after digestion [47, 48]. Tracking oxidation across a gradient of denaturant concentrations yields protein folding stability profiles: ligand binding can stabilize a protein by shifting methionine exposure to higher denaturant levels, or destabilize it by accelerating unfolding and shifting oxidation to lower levels. These spectral shifts reflect the net energetic impact of ligand binding (Figure 2A).

Although developed for soluble proteins, SPROX has also been applied to membrane‐associated targets. For example, Geer and Fitzgerald used it to map the adenosine triphosphate (ATP) interactome in yeast, identifying 28 ATP‐sensitive proteins, including membrane‐associated proteins such as tPMA1 (H^+^‐ATPase) and VMA1 (V‐ATPase) [23]. Using SPROX with isobaric‐tag labeling, multiple samples could be analyzed in a single MS run, allowing direct comparison of protein stability in different conditions and revealing both stabilization and destabilization effects, reflecting direct ligand binding as well as allosteric and broader conformational changes. However, despite the demonstrated sensitivity, MPs present a particular challenge for SPROX, as transmembrane helices and lipid‐embedded sequences often contain few accessible methionine residues. The coverage is therefore limited when methionines are sparse, buried, or sequestered within the membrane bilayer [49].

### 3.2 Thermal Stability Methods

#### 3.2.1 The Cellular Thermal Shift Assay

The CETSA measures ligand‐induced changes in protein thermal stability. Upon ligand binding, this thermal stability shifts, typically upward, offering an indirect but quantifiable measure of target engagement. Following ligand exposure, cells or lysates are subjected to a temperature gradient. Proteins that remain folded after heating stay in the soluble fraction, while denatured proteins aggregate. The soluble fraction is then quantified by Western blot, enzyme‐linked immunosorbent assay (ELISA), or differential scanning fluorimetry (DSF). DSF uses an environment‐sensitive fluorescent dye that binds to the hydrophobic surface exposed during protein unfolding, generating melting curves whose midpoints indicate the protein's melting temperature (*T*_m_). Ligand binding typically increases *T*_m_, providing a readout of stabilization [7, 50]. Together, DSF and CETSA are widely used in drug discovery to quantify ligand–protein interactions across diverse affinities [11, 50, 51]. The method can be applied to intact cells, lysates, or tissue extracts to assess target engagement in native‐like environments [51]. CETSA also supports an isothermal dose–response format, where thermal stabilization is measured at a single temperature with variable ligand concentration, generating dose–response curves which are often used as an orthogonal approach to estimate half maximal inhibitory concentration (IC_50_) or *K*_d_ values.

A key advantage of CETSA is its simplicity: it requires no proteolytic digestion, MS instrumentation, or specialized data analysis. The method is adaptable to various MP classes, including SERCA2, PAR2, MCT4, and the adenosine A_2_A receptor [30, 52, 53]. Despite these advantages, the use of CETSA for MPs is limited by biochemical constraints of maintaining ligand–protein interactions after solubilization, plus the need for validated antibodies. These factors restrict assay scalability and target breadth, and contribute to the persistent under‐representation of MPs in CETSA datasets [7, 11, 30, 54].

#### 3.2.2 Thermal Proteome Profiling

TPP overcomes the throughput limitation of CETSA by integrating MS‐based proteomics [55, 56]. Like CETSA, TPP applies a temperature gradient to intact cells or lysates treated with ligands, inducing protein unfolding (Figure 2B). Soluble fractions are digested, optionally tandem mass tag (TMT)‐labeled, and analyzed by LC‐MS/MS, enabling proteome‐wide thermal shift curves for broad detection of direct and indirect ligand targets. TPP also captures off‐target interactions in complex proteomes, offering insights into drug specificity and safety [57]. Workflow variants, such as compound titration TPP and 2D‐TPP, systematically assess thermal shifts across drug concentration and temperature–concentration gradients, allowing more sensitive characterization of protein–ligand interactions [54]. Cell‐surface TPP (CS‐TPP) represents a specialized adaptation of TPP for MPs, achieved by enriching glycosylated surface proteins with hydrazide beads that capture oxidized sialic acids. This approach enables ligand‐induced stabilization to be detected in intact membrane contexts, as demonstrated for CCR5 and the transporters MCT1 and MCT3, thereby addressing challenges that conventional TPP faces with MPs [13].

TPP has been widely applied to soluble proteomes across diverse cell types, making it a cornerstone of proteomics and drug discovery [7, 58]. However, its reliance on protein abundance and MS ionization efficiency hampers the detection of low‐expression or poorly ionizing proteins, particularly MPs. Nonetheless, TPP has successfully profiled MPs such as CD45 and the Na⁺/K⁺‐ATPase α‐subunit's thermal stabilization in response to incubation with ouabain [11, 13, 59]. However, like CETSA, TPP relies on precise stability signatures, and detergent‐induced variability can disrupt protein folding, alter lipid–protein interactions, or introduce nonspecific stabilization effects that obscure genuine ligand‐induced shifts. To mitigate these challenges, experiments often incorporate matched controls and target‐specific optimizations, for instance, stabilizing GPCRs with cholesterol hemi‐succinate or applying cell surface capture (CSC) to enrich glycosylated surface MPs [13, 60, 61]. These limitations, along with strategies that have been developed to overcome them, are discussed in detail in Section 5 of the review.

Despite the strengths, TPP remains limited by labor‐intensive sample preparation. Experiments require control and ligand‐treated samples across multiple temperatures, with biological triplicates for reproducibility. Dozens of samples are needed because stabilization often appears only at specific temperatures, necessitating full denaturation curves rather than single‐point measurements [55, 62]. To streamline workflows, George et al. treated acute myeloid leukemia cells with losmapimod and compared tandem mass tag‐based data‐dependent acquisition (TMT‐DDA) and label‐free data‐independent acquisition (DIA) within TPP, finding that TMT‐DDA identified more targets while DIA gave more consistent quantification [63]. Ball et al. introduced the isothermal shift assay (iTSA), measuring solubility at a single temperature with replicates, nearly doubling kinase detection despite losing full thermal curve resolution. More recently, Zijlmans et al. developed single‐tube TPP with uniform progression (STPP‐UP), reducing handling via a one‐tube temperature ramp; although less sensitive, it provides a practical high‐throughput option for early drug screening [62, 64]. Together, these continuous methodological innovations and protocol optimizations underscore the growing potential of TPP not only for soluble proteomes but also for systematic, high‐throughput exploration of MPs in pharmacological research.

### 3.3 Proteolytic Resistance Methods

Drug affinity responsive target stability (DARTS) exploits ligand‐induced stabilization, using protease protection and gel‐based separation to identify targets [65, 66]. Although effective for soluble proteins, DARTS is less suitable for MPs due to limited protease access to transmembrane domains [49, 67]. More recently, MS‐based limited proteolysis (LiP‐MS) has extended this concept by mapping ligand‐induced proteolytic changes at the peptide level [68, 69]. LiP‐MS workflows incubate protein lysates with small molecules, apply broad‐spectrum proteases, and analyze resulting cleavage patterns by LC‐MS/MS (Figure 2C). Ligand binding alters protease accessibility, revealing ligand–protein interactions. Soste et al. demonstrated this by detecting conformational changes in the GPCR ACKR3, showing that complex MPs can be probed without prior structural knowledge [70]. Similarly, proteome‐wide elution profile learning for small‐molecule activity (PELSA) combined limited proteolysis with SEC to map the binding site of the kinase inhibitor lapatinib on ERBB2, increasing spatial resolution by linking protease accessibility shifts to distinct SEC fractions, thereby narrowing the binding event to specific protein regions [71]. Most recently, thermostability‐assisted LiP‐MS (TALiP‐MS) improved sensitivity by enriching thermally stabilized proteins before proteolysis: drug‐treated lysates are heated, centrifuged, and heat‐denatured proteins removed. This approach increased target peptide detection by up to eightfold [72]. Although not yet applied to MPs, TALiP‐MS offers a promising route for extending proteolytic profiling to challenging protein classes. We provide a summary of affinity‐ and stability‐based and their applications to MPs in Table 1.

## 4 Emerging MS Methodologies for Characterizing Ligand‐MP Interactions

MS methodologies for detecting ligand–MP interactions are frequently hindered by the separation problem: detergents, fractionation, or extensive sample preparation that can disrupt native assemblies, causing ligands, cofactors, or lipids to dissociate from proteins, preventing the detection of transient interactions. This is a major drawback for drug discovery, since many small‐molecule therapeutics act through transient, reversible binding [73, 74].

Native MS addresses this challenge by analyzing intact proteins and complexes in their non‐denatured form, transmitting whole assemblies into the gas phase while preserving noncovalent interactions. This enables direct measurement of ligand stoichiometry as well as co‐bound lipids and cofactors [32]. For example, Yen et al. showed that native MS detects PIP_2_ bound in discrete stoichiometries (e.g., one or two molecules per receptor) to class A GPCRs (β_1_‐adrenergic receptor, A_2_A receptor, and neurotensin receptor 1), stabilizing their active receptor‐G–protein complexes and directly linking lipid occupancy to functional states [75].

Nativeomics extends native MS by coupling intact protein analysis with tandem MS (MS/MS) to directly identify ligands. Here, intact protein–ligand assemblies are measured (MS1), then mass‐selected to exclude free ligands. Collisional dissociation releases bound molecules while keeping the protein complex intact, after which the liberated ligand is fragmented (MS2) and matched to reference spectra. This confirms both ligand identity and its direct association with the protein [33]. For instance, Gault et al. applied nativeomics to the porin OmpF and directly identified its transient peptide ligand OBS1, demonstrating chemical identification of ligands in preserved MP–ligand complexes [33].

Another complementary strategy is to analyze protein assemblies ejected directly from native or reconstituted membranes. In this approach, crude membranes, either native or reconstituted, are introduced into the mass spectrometer, without detergent solubilization, through a narrow capillary that disperses the liquid into microscopic droplets. As the droplets evaporate, intact membrane protein (MP) complexes are released from the membrane together with their bound lipids, enabling direct MS analysis. Chorev et al. demonstrated this with the AcrAB–TolC efflux pump directly ejected from *Escherichia coli* membranes, showing cobound phospholipids and highlighting cardiolipin as a key stabilizer of the assembly [34].

In parallel, advances in MS‐based solution thermochemistry and IM‐MS have expanded the resolution of MP–ligand interactions. Thermochemical MS destabilizes complexes through controlled collisional activation or temperature ramps, measuring unfolding or dissociation thresholds that shift upon ligand binding, conceptually similar to TPP [35]. For example, Yen et al. showed that agonists stabilized the β_1_‐adrenergic receptor–G protein complex against activation, whereas antagonists had little effect or destabilized it [76]. IM‐MS, by contrast, measures collision cross sections (CCSs) to distinguish compact from extended conformations and to track ligand‐induced shifts in conformational ensembles. Such shifts are informative because they show how ligands bias the conformational ensemble, either stabilizing functional states or pushing proteins toward less active and more heterogeneous states [77]. Marcoux et al. demonstrated that cyclosporin A binding to P‐glycoprotein ABCB1 promoted compact, active‐state conformations, highlighting how IM‐MS can directly reveal drug‐induced modulation of MP structure [78].

These MS‐based advances complement progress in detergents and MMs, and their integration is likely to further expand the range of accessible systems. Ongoing developments are expected to broaden MS applications to complex proteomes, accelerating MP–ligand discovery and expanding opportunities in drug development.

## 5 Shortcomings of Detergent in Membrane Proteins‐Ligand Screenings

Although detergents are highly effective for extracting MPs and remain essential in many workflows, they often struggle to fully replicate the physiological environment. The disruptive effects of detergents on MP capture are well documented. Kawatkar et al. used the non‐ionic detergent NP‐40 in thermal stability assays to targets such as TSPO, SERCA2, and PAR2, but reported substantial variability: conditions required manual optimization for each target, and thermal shift signatures were often weak or inconsistent, especially for GPCRs and low‐affinity ligands [30]. Some challenges stem not only from protein instability but also from detergent behavior. Berlin et al. demonstrated that detergent micelles approaching their cloud point—where they phase‐separate into detergent‐rich and detergent‐poor phases—can compromise protein solubility and distort thermal shift measurements, leading to aggregation or artifacts unrelated to true unfolding [14]. Interestingly, Brown et al. showed that the same cloud‐point phenomenon can be exploited for protein enrichment: using non‐ionic detergents that phase‐separate upon heating, they applied cloud‐point extraction to drive hydrophobic proteins into the detergent‐rich phase, selectively enriching MPs. This strategy enabled detection of low‐abundance, highly hydrophobic proteins, including endogenous MPs with up to 19 transmembrane domains that are often missed in conventional proteomic workflows [79]. Recognizing the challenges posed by detergents in thermal assays, Ye et al. introduced a modified, lower‐temperature TPP workflow. They demonstrated that NP‐40 and zwitterionic CHAPS detergents induce protein aggregation above 50°C, obscuring ligand‐induced stabilization, whereas reducing the thermal window restored melting shifts and revealed MP–ligand interactions otherwise undetected by standard TPP [31]. Together, these studies highlight how detergent phase behavior can both hinder and be harnessed in MP analysis, and how optimization or alternative chemistries are needed to reduce artifacts while enabling broader applicability.

Urner et al. first introduced oligoglycerol detergents (OGDs), a modular class that improved charge‐state resolution in native MS, preserved bound lipids, and maintained agonist binding of receptors such as NTSR1 [80]. Building on this, Urner et al. developed hybrid detergents with tunable hydrophilic–lipophilic balance, revealing lipopolysaccharide binding to bacterial inner MPs, including modulation of the transporter BtuCD [81]. Chang et al. developed hetero‐bicephalic detergents with tunable dual headgroups that lower average charge states and preserve labile MP assemblies, as demonstrated with two distinct systems: tetrameric AqpZ and a GPCR–nanobody complex [36]. At the proteome scale, Latosinska et al. reviewed advances in detergent design, highlighting how tailored architectures improve MS compatibility by enhancing protein stability, reducing charge states, and enabling more reliable detection of intact MP assemblies [82]. In a related review, Lee et al. described pendant‐bearing detergents, in which short alkyl side chains branch from the main detergent tail. These pendants improve MP stability by strengthening hydrophobic packing, reducing micelle hydration, and tuning micelle properties to pendant length and number [83]. Finally, acid‐labile surfactants such as RapiGest provided one of the first detergent‐compatible MS workflows. They solubilize MPs during sample preparation but degrade under acidic conditions, allowing detergent removal before MS analysis and avoiding the ion suppression caused by detergents. They demonstrated this by identifying multi‐pass transporters, including LacY and the PstA/PstC components of the phosphate transport system, highlighting the feasibility of recovering highly hydrophobic MPs for MS analysis [84].

Together, these advances highlight how creative detergent design continues to expand the analytical window for MPs. Importantly, recent work has demonstrated that improved detergent formulations can be directly integrated into proteomics workflows, enhancing the solubilization, stability, and detection of MPs in *omics*‐based analyses. These innovations show that detergents not only remain indispensable for MP research but also continue to evolve as versatile tools that complement emerging MM systems. At the same time, no reconstitution system is entirely inert: interactions with proteins, lipids, and ligands can complicate interpretation, particularly in assays sensitive to subtle conformational shifts. These challenges have motivated the parallel development of protein/peptide‐ and polymer‐based MMs, which provide complementary strategies for expanding the reach of MP–ligand studies.

## 6 Implementing Membrane Mimetics in Membrane Protein‐Ligand Screens

Alongside detergents, MMs have emerged as valuable alternatives, providing a means to study MPs in aqueous environments while retaining native‐like environments. Platforms such as nanodiscs, peptidiscs, and SMA lipid particles (SMALPs) recreate bilayer‐like environments in vitro, offering controlled conditions that maintain protein stability and functional fidelity. Although most successfully leveraged in structural biology, these MMs are now being adapted for use in functional assays, including MS‐based ligand screening, where they show potential to preserve MP–ligand interactions [85, 86, 87]. Accordingly, recent proof‐of‐concept studies indicate that such systems can expand the analytical reach of drug discovery by making MPs more tractable to proteomics‐based, high‐throughput workflows [10, 88, 89, 90, 91, 92, 93, 94, 95, 96, 97, 98, 99, 100].

### 6.1 Nanodiscs for Proteome‐Level Analysis and Screening

**FIGURE 3:**
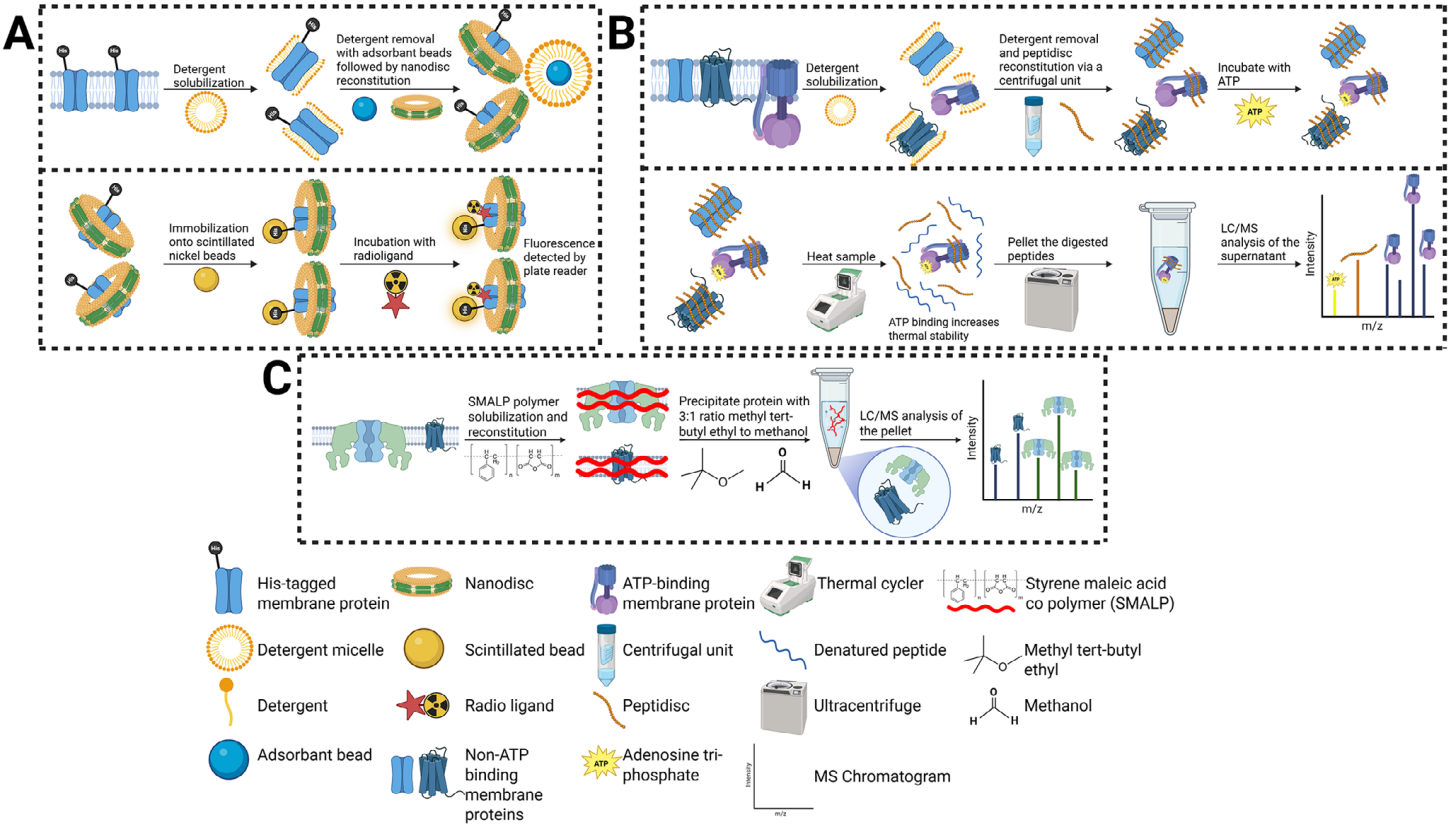
Membrane mimetic strategies for membrane protein ligand interaction studies and for membrane proteome profiling. (A) Schematic of nanodisc scintillation proximity assay (SPA). A His‐tagged membrane protein (MP of interest is solubilized in detergent and reconstituted into nanodiscs following detergent removal with adsorbent beads. The nanodisc‐reconstituted MP is immobilized on scintillation‐coated beads via a His‐tag. A radiolabeled ligand is then incubated with the immobilized nanodisc‐MP. Upon binding to the MP, proximity to the scintillant surface enables detection of ligand–protein interactions via emitted light. (B) Schematic of membrane mimetic thermal proteome profiling (MM‐TPP). MPs are solubilized in detergent and reconstituted with peptidisc using a centrifugal unit to remove detergent and facilitate peptidisc self‐assembly around MPs. The reconstituted membrane proteome is incubated with or without a ligand (ATP), then subjected to heat‐induced denaturation using a thermal cycler. Following ultracentrifugation, thermally unstable proteins pellet, while stabilized proteins remain in the supernatant. Peptides from the pellet or supernatant are digested and analyzed by LC/MS. Ligand‐induced thermal stabilization is inferred from shifts in the abundance of specific MPs between control and ligand‐treated conditions. (C) Schematic of styrene maleic acid lipid particle (SMALP) membrane proteome profiling. MPs are directly extracted from crude membrane preparations using styrene–maleic acid (SMA) copolymer, which solubilizes MPs in lipid bilayers. To separate captured MPs from the SMA copolymer, the solubilized proteome is subjected to precipitation using a 3:1 ratio of methyl tert‐butyl ether and methanol. This results in the SMA copolymer and lipids remaining in the supernatant, while MPs are pelleted. The protein pellet is then digested and analyzed via LC/MS.

Nanodiscs are discoidal lipid bilayers stabilized by two amphipathic MSPs, typically derived from apolipoprotein A1. Initially, MPs are solubilized with detergent followed by detergent removal via adsorbent beads. MSPs self‐assemble with phospholipids and MPs into soluble assemblies, allowing for tunable assembly diameter size or tailoring of lipid composition. This modularity makes nanodiscs particularly valuable for dissecting ligand–MP interactions [86, 89, 101]. For example, nanodiscs have facilitated detailed analysis of substrate binding to cytochrome P450 enzymes, supporting precise kinetic measurements of drug metabolism and allosteric regulation [101]. Additionally, they have enabled mechanistic studies of receptors such as the calcium‐sensing receptor (CaSR) and β2‐adrenergic receptor (β2‐AR), allowing detailed investigation of ligand binding and G protein activation mechanisms at their interfaces [88]. Beyond structure, nanodiscs have supported a wide range of biochemical assays for MPs, including scintillation proximity assays (SPAs) for MP–ligand binding (Figure 3A) and fluorescence‐based activation assays [89, 90, 91].

Despite these advantages, broader adoption of nanodiscs for high‐throughput MS applications has lagged. Originally designed for tailored reconstitution of individual MPs, they may be less well suited for unbiased large‐scale protein capture, and only a few studies have explored their use in proteome‐wide contexts. Still, their versatility and ability to mimic native‐like lipid environments highlight their promise as a platform that could be further adapted for systematic MP‐focused investigations.

Early work by Marty et al. reported incorporation efficiencies of up to ∼85% for *E. coli* MPs into nanodiscs [92]. Wilcox et al. extended this library approach to synaptosomal membranes, successfully preserving receptor activity as confirmed by functional assays. Nanodisc libraries were further integrated with MS to identify Alzheimer‐associated amyloid‐β oligomer (AβO) targets: UV‐crosslinking of AβOs to MPs enabled affinity purification and peptide‐level identification by LC‐MS/MS. The same platform also supported small‐molecule screening, leading to the discovery of aurin tricarboxylic acid, which reduced AβO binding by over 90% in SMPL rat hippocampal neurons [93]. Building on these advances, Mak et al. streamlined nanodisc assembly by working directly from whole‐cell lysates, enabling rapid generation of MP‐enriched libraries from sources such as HEK293 and red blood cells [94]. Similarly, Roy et al. optimized GPCR solubilization from osteosarcoma cells by adjusting lipid composition with anionic lipids and cholesterol [95]. Collectively, these studies illustrate how nanodiscs are moving beyond structural biology toward functional screening, with growing potential to link membrane proteomics and drug discovery.

### 6.2 Other Emerging Membrane Mimetic Platforms for Membrane Protein Screening

The peptidisc platform builds on the same self‐assembly principle as nanodiscs but replaces scaffold proteins with amphipathic peptides that wrap around MPs to maintain them in aqueous solution. Following detergent solubilization of native membranes, these peptides spontaneously assemble around MPs, providing a robust and scalable method for detergent‐free stabilization. Unlike nanodiscs, peptidiscs adopt a “one‐size‐fits‐all” architecture, allowing straightforward reconstitution of a wide spectrum of MPs [85].

A notable advancement is the His‐tagged peptidisc, developed by Young et al., which enables downstream purification of the membrane proteome via nickel‐affinity chromatography [96]. Initially applied in *E. coli*, the His‐tagged peptidisc resulted in an ∼20% enrichment of MPs and an ∼35% depletion of non‐membrane components based on MS analysis, compared to a peptidisc library prepared without nickel‐affinity enrichment. Subsequent studies extended the platform to more complex systems: Zhao et al. successfully reconstituted MPs from HeLa cells, while Antony et al. achieved proteome‐wide stabilization in mouse liver tissue, collectively identifying over 800 unique MPs [97, 98, 99]. Building on this foundation, Jandu et al. introduced membrane mimetic‐thermal proteome profiling (MM‐TPP), integrating peptidisc libraries into a TPP screening format (Figure 3B). Using *E. coli* and mouse liver proteomes, they profiled ATP‐ and orthovanadate‐induced stabilization of ATP‐binding MPs, including ABC transporters, while also detecting off‐target effects linked to ADP‐ and AMP‐binding proteins from ATP metabolism [10]. Notably, DDM solubilization with SP4 cleanup failed to capture these stabilization events, whereas MM‐TPP successfully detected specific ligand binding, such as 2‐MeS‐ADP stabilizing P2RY12. These findings highlight MM‐TPP's ability to distinguish specific from nonspecific interactions and demonstrate the feasibility of peptidisc‐based libraries for proteome‐scale ligand discovery in MPs.

A third, emerging MM system is the SMA copolymer, which extracts MPs by solubilizing intact nanoscale membrane fragments termed SMALPs, eliminating the need for detergents entirely [89, 102, 103]. Although mostly applied for structural studies, SMALPs have also been adapted for proteomics and ligand screening workflows. For example, Sharma et al. showed that SMALP‐reconstituted tetraspanins such as CD81 and CD53 retained epitope accessibility and ligand‐binding kinetics comparable to those in intact membranes, as confirmed by surface plasmon resonance [100, 104].

However, challenges remain. Kamilar et al. reported that SMALPs can exhibit heterogeneous morphologies, forming assemblies ranging from single particles to mixed micelles; SMA–lipid assemblies with differing sizes and compositions [87]. In addition, SMALPs may interfere with transmembrane domains or introduce chemical artifacts during MS analysis [87, 105, 106]. These issues are particularly relevant for ligand‐binding studies: interactions may be missed if MPs are structurally compromised, SMA obstructs access to buried transmembrane sites, or variability in SMALP morphology yields inconsistent outcomes. Nevertheless, SMALPs are increasingly useful for proteome‐scale applications. Mueller et al. benchmarked SMA variants (200, 300, 502‐E) against detergents such as DDM and lauryl maltose neopentyl glycol (LMNG) in HEK293 cells, showing equal or superior extraction across diverse MP classes [107]. More recently, Brown et al. applied tailored formulations to generate libraries of over 2000 mammalian MPs fully compatible with MS‐based workflows (Figure 3C) [26].

Together, these emerging platforms provide new opportunities for linking MPs with MS‐based ligand discovery. Peptidiscs provide scalability and affinity enrichment, while SMALPs circumvent the need for detergents. Both approaches are expanding the toolkit for probing ligand–protein interactions. Recently, Antony et al. compared solid‐state cleanup, peptidisc, nanodisc spNW30, and SMA2000 for profiling the mouse liver membrane proteome and assessing MP‐level dysregulation. In this study, the peptidisc showed higher proportional enrichment of MPs and improved detection of dysregulation between healthy and obese mouse liver [40]. Further work will be needed to evaluate how different MMs retain native‐like environments and capture ligand–MP binding. Collectively, these technologies are broadening experimental options for making MPs more accessible to high‐throughput screening and functional analysis.

### 6.3 Recent Advances in Detergent‐Free Polymer‐Free Systems

Nanodiscs and peptidiscs require an initial detergent step to extract MPs, a process that can displace native lipids or perturb protein structure, potentially affecting ligand‐binding sites. Although they do not eliminate detergents, these systems provide a more stabilizing environment than detergent micelles, with reconstituted proteins often retaining higher activity and ligand binding [108, 109, 110]. Importantly, workflows such as MM‐TPP have demonstrated that these scaffolds are fully compatible with MS‐based analyses [10]. Building on these advances, SMA copolymers enable direct detergent‐free solubilization into SMALPs. Although SMALP‐reconstituted MPs have been shown to preserve ligand binding and conformational response, their use in high‐throughput or systematic ligand‐screening workflows remains underexplored [111, 112]. However, a fundamental biophysical limitation shared by all MMs is that they are supramolecular assemblies in which proteins, lipids, and ligands rapidly exchange through collisional processes in solution [113]. To overcome these constraints, the next‐generation detergent‐free systems we term “Peptergents” are emerging, such as 4F‐based peptidiscs and detergent‐free nanodiscs (DeFrNDs) use engineered scaffold peptides to directly extract MPs without the use of detergents [114, 115].

These proof‐of‐principle studies highlight both the opportunities and trade‐offs of these approaches. For example, the 4F peptidisc achieved ∼40% of the solubilization efficiency relative to LMNG when reconstituting hTRPC3 in Sf9 insect cells, yet preserved the activity of the NOX2–p22 complex, which was lost in detergent micelles [114]. Similarly, Ren et al. showed that MalFGK2, an ABC transporter whose basal ATPase activity is normally low and tightly coupled to maltose transport in proteoliposomes, became uncoupled and hyperactive when solubilized in detergent but retained its regulated activity when reconstituted in DeFrNDs [115]. Taken together, current evidence suggests that detergent‐free systems trade solubilization efficiency for improved preservation of protein structure and function. How these trade‐offs position them within the broader landscape of MMs and MS‐based ligand discovery remains uncertain, highlighting the need for further systematic evaluation.

## 7 Conclusion and Future Perspectives

Despite their central role in cellular function, MPs have historically been underrepresented in ligand screening campaigns, largely due to their low abundance, structural complexity, poor trypsin digestion, and incompatibility of detergents with MS. However, recent innovations are helping to overcome these challenges by providing strategies that better preserve native MP structure and function. MS, long established for soluble protein analysis, is now being extended to applications for membrane proteomics and MP–ligand discovery.

Advances in MS strategies, including native MS, nativeomics, native/reconstituted membrane ejection, innovations in detergent chemistry and MS‐compatible detergents, the development of detergent‐free MM platforms, and the convergence of MMs with affinity‐ and stability‐based MS, offer the enticing prospect of improving MP–ligand screening and driving their systematic integration into high‐throughput workflows. Furthermore, pairing direct binding strategies such as AS‐MS with activity‐based assays further strengthens drug discovery pipelines by not only identifying ligand binding events but also establishing mechanistic links to functional outcomes [116].

Looking ahead, continued refinement and integration of these approaches will be critical. Although the field is still in its early stages, the progress highlighted here establishes both feasibility and momentum, underscoring the clear potential for systematic MP–ligand discovery. Together, these advances are shaping an emerging framework in which MPs can be routinely targeted, laying the groundwork for future breakthroughs in both drug discovery and mechanistic understanding.

## Conflicts of Interest

The authors declare no conflicts of interest.

## Data Availability

The authors have nothing to report.
